# Effectiveness of laser pulsed irradiation for antimicrobial photodynamic therapy

**DOI:** 10.1007/s10103-024-04103-1

**Published:** 2024-06-05

**Authors:** Kentaro Sueoka, Taiichiro Chikama, Koichiro Shinji, Yoshiaki Kiuchi

**Affiliations:** 1 Department of Ophthalmology and Visual Science, Graduate School of Biomedical Sciences, Hiroshima University, 1-2-3 Kasumi, Minami-Ku, Hiroshima, 734-8551 Japan; 2 Department of Ophthalmology, Hiroshima Prefectural Hospital, 1-5-54 Ujinakanda, Minami-Ku, Hiroshima, 734-8530 Japan

**Keywords:** Antimicrobial photodynamic therapy (aPDT), Light-emitting diode (LED), Laser diode (LD), Pulsed irradiation, *Staphylococcus aureus*

## Abstract

The aim of this study was to compare two types of light irradiation devices for antimicrobial photodynamic therapy (aPDT). A 660-nm light-emitting diode (LED) and a 665-nm laser diode (LD) were used for light irradiation, and 0.1 mg/L TONS 504, a cationic chlorin derivative, was used as the photosensitizer. We evaluated the light attenuation along the vertical and horizontal directions, temperature rise following light irradiation, and aPDT efficacy against *Staphylococcus aureus* under different conditions: TONS 504 only, light irradiation only, and TONS 504 with either LED (30 J/cm^2^) or LD light irradiation (continuous: 30 J/cm^2^; pulsed: 20 J/cm^2^ at 2/3 duty cycle, 10 J/cm^2^ at 1/3 duty cycle). Both LED and LD light intensities were inversely proportional to the square of the vertical distance from the irradiated area. Along the horizontal distance from the nadir of the light source, the LED light intensity attenuated according to the cosine quadrature law, while the LD light intensity did not attenuate within the measurable range. Following light irradiation, the temperature rise increased as the TONS 504 concentration increased in the order of pulsed LD < continuous LD < LED irradiation. aPDT with light irradiation only or TONS 504 only had no antimicrobial effect, while aPDT with TONS 504 under continuous or pulsed LD light irradiation provided approximately 3 log reduction at 30 J/cm^2^ and 20 J/cm^2^ and approximately 2 log reduction at 10 J/cm^2^. TONS 504-aPDT under pulsed LD light irradiation provided anti-microbial effect without significant temperature rise.

## Introduction

Infectious keratitis can result in corneal perforation or scarring and is one of the major causes of blindness worldwide [1]. Antibiotic agents are currently the most reliable and widely accepted treatment for infectious keratitis. However, the overuse of broad-spectrum antibiotics without an appropriate diagnosis of bacterial and fungal keratitis accelerates antimicrobial resistance [2–4]. Therefore, alternative approaches for combating infectious keratitis are urgently required.

Photodynamic therapy (PDT) has been developed to destroy cancer cells or to induce regression of new blood vessels by the action of reactive oxygen species generated through the interaction of irradiation light of a specific wavelength with a photosensitizer accumulated in target cells [5, 6]. This approach has been applied to treat various types of cancer [5] and age-related macular degeneration [7]. PDT against microorganisms dates back to 1900 with the observed cytotoxicity of paramecia exposed to sunlight in the presence of an acridine dye [8]. The application of PDT to microorganisms declined, however, after the introduction of antibiotics, and the focus of PDT switched to cancer treatment [9, 10]. The recent appearance of drug-resistant bacteria [11–13] has led to renewed interest in the antimicrobial action of PDT, which is now known as antimicrobial photodynamic therapy (aPDT) [14].

We have been investigating the combination of a newly developed photosensitizer called TONS 504 and light irradiation as potential aPDT of corneal infections. In our previous studies, we showed that TONS 504 provides a higher singlet oxygen quantum yield and photodynamic antimicrobial effect than methylene blue, which is widely used as a photosensitizer for aPDT [15]. Additionally, we showed that aPDT delivered with this new photosensitizer (TONS 504–aPDT) induces in vitro inactivation of several pathogens, including bacteria, viruses, fungi, and protozoa, especially drug-resistant bacteria causing corneal infections [16–20]. Furthermore, TONS 504–aPDT is effective against a model of acanthamoeba keratitis in vivo [21].

In PDT on cancer tissue, it has been reported to promote an effective photoresponse with tissue heating [22]. PDT under strong conditions produces high antimicrobial efficacy, but in the infectious keratitis we are targeting, it may have adverse effects on normal tissue, such as reduced corneal transparency due to protein denaturation. Therefore, in this study, we investigated the effect of the light irradiation method on the anti-microbial efficacy of TONS 504–aPDT. Specifically, we compared the characteristics of light emitted by two devices: a new laser diode (LD) system and a conventional light-emitting diode (LED) system. In addition, we measured the temperature rise following light irradiation and evaluated the antimicrobial efficacy of aPDT under different conditions: TONS 504 only, light irradiation only, and TONS 504 with either LED (30 J/cm^2^) or LD light irradiation (continuous vs. pulsed).

## Material and methods

### Microorganisms

A strain of *Staphylococcus aureus* (ATCC 25923) obtained from NITE Biological Resource Center was grown in liquid medium overnight at 37 °C inside a shaking incubator. The liquid medium consisted of polypepton (Nihon Pharmaceutical Company, Tokyo, Japan), dried yeast extract, and magnesium sulfate heptahydrate (Nacalai Tesque, Kyoto, Japan). The cells were harvested by centrifugation (3000 × *g* for 10 min at 4 °C), washed three times with phosphate-buffered saline (PBS), and suspended in PBS.

### Photosensitizer

The hydrophilic and cationic chlorin derivative TONS 504 [13,17-bis(1-carboxyethyl)carbamoyl(3-methylpyridine)-3-(1,3-dioxane-2-yl)methylidene-8-ethenyl-2-hydroxy-2,7,12,18-tetramethyl chlorin, diN-methyl iodide (C51H58N8O5I2)], a dark green powder with a molecular weight of 1116.9, was obtained from Porphyrin Laboratory (Okayama, Japan). It was dissolved and diluted to several concentrations with PBS.

### Light irradiation device

**Fig. 1:**
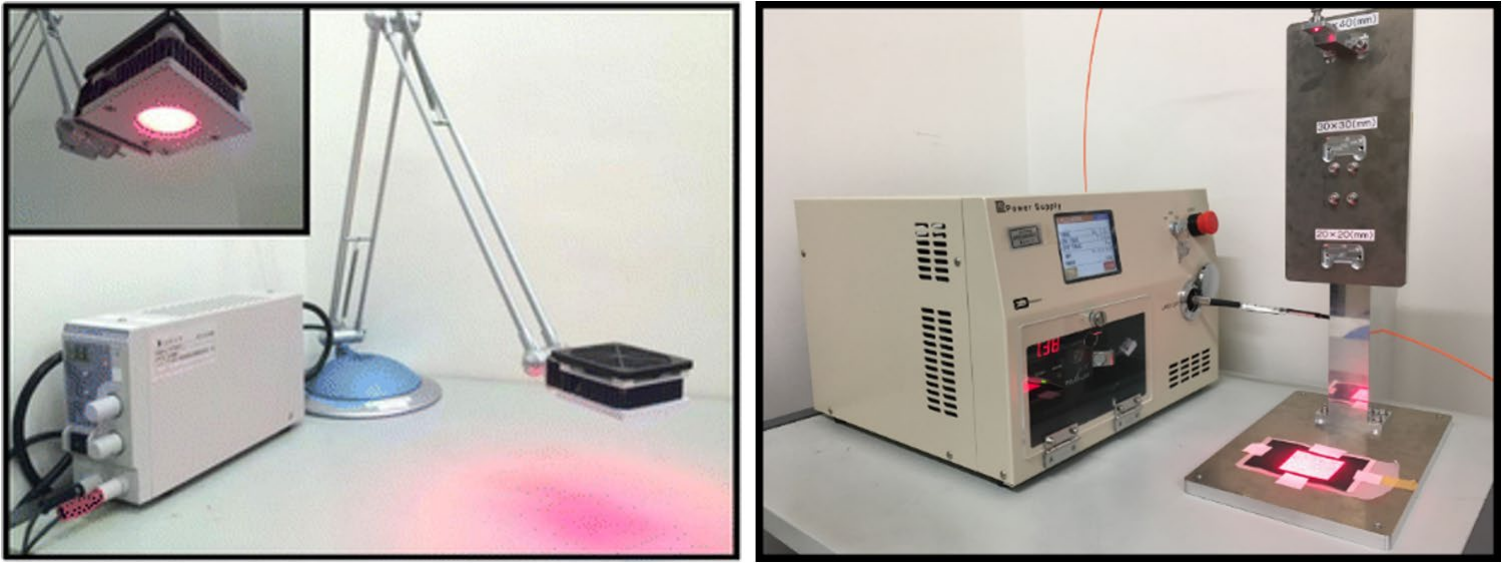
(left) Conventional LED system and (right) LD system with pulsed irradiation mode

A conventional LED system (ME-PT-DSRD660–0201), its light sources is a matrix of LEDs delivering light centered at the wavelength of 660 nm (Fig. 1) was obtained from CCS (Kyoto, Japan). In this study, a LD system (PCTH 195) capable of pulsed and continuous irradiation and emitting light at the wavelength of 665 nm (Fig. 1) was obtained from UNITAC (Hiroshima, Japan). The light power was measured with an optical power meter (Hioki, Nagano, Japan). Vertical light attenuation was measured at 25 to 200 mm from the surface irradiated by the LED light device and at 157, 247, and 327 mm from the area irradiated by the LD light device. In addition, horizontal light attenuation was measured at 0 to 50 mm from the area irradiated by the LED device and within the irradiation field of the LD device owing to its high light linearity. The increase in temperature following irradiation was measured with a wire thermometer placed in a 24-well plate containing TONS 504 diluted to various concentrations. The vertical height was irradiated from 50 mm for the LED light device and from 327 mm for the LD light device.

### aPDT

**Fig. 2:**
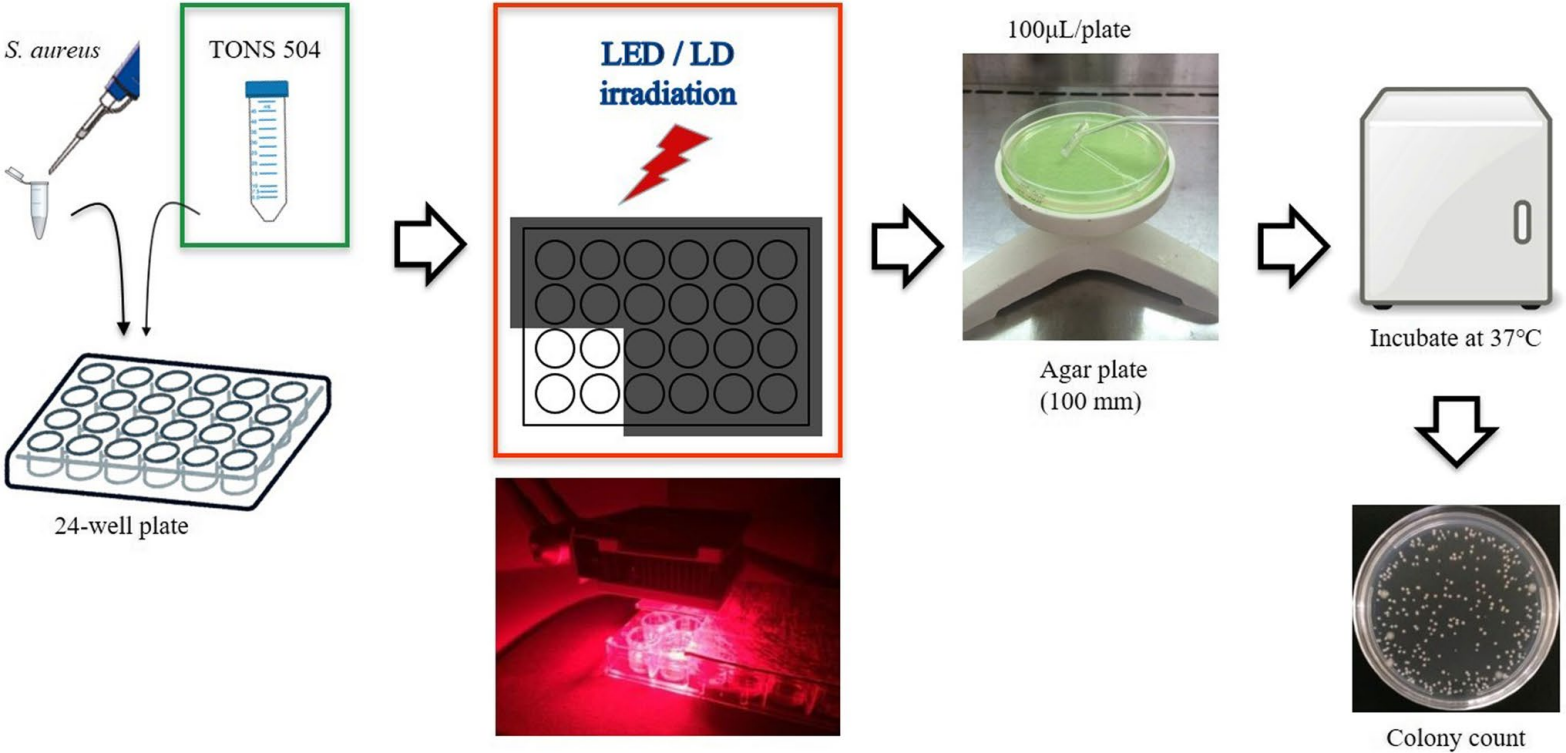
Experimental overview and workflow

The experimental overview and workflow are shown in Fig. 2. First, the differences in the antimicrobial photodynamic effects of LED and LD were evaluated at the same 30 J/cm2 of continuous irradiation exposure. The vertical height was irradiated from 50 mm for the LED light device and from 327 mm for the LD light device. No light irradiation was set as a control, and TONS504 concentrations were assigned to 0, 0.01, 0.1, 1, and 10 mg/L.

Next, the differences in the antimicrobial photodynamic effect of the irradiation mode in the LD light device were examined. The TONS504 concentration was 0.1 mg/L and light irradiated from a vertical height of 327 mm. Pulsed LD irradiation was delivered at 2/3 duty cycle (equivalent to 20 J/cm^2^ of total light irradiation) and 1/3 duty cycle (equivalent to 10 J/cm^2^ of total light irradiation).

Bacteria and TONS 504 in a total volume of 1 mL were placed in the wells of a 24-well plate and then incubated for 5 min at room temperature before LED or LD irradiation. After light irradiation, 100 μL of the contents of a well (1 × 10^8^ colony-forming units (CFU) of bacteria) was transferred to an agar plate (100 mm in diameter, containing agar [Nacalai Tesque] in liquid medium). The agar plates were incubated at 37 °C, after which visible colonies were counted.

### Statistical analysis

Quantitative data were analyzed for statistically significant relationships among groups with one-way analysis of variance (ANOVA) followed by Dunnett’s post hoc test. Results with P-value < 0.01 were considered statistically significant. Statistical analysis was performed with JMP software version 16.2.0 (SAS Institute, Cary, NC, USA).

## Results

### Light intensity

**Fig. 3:**
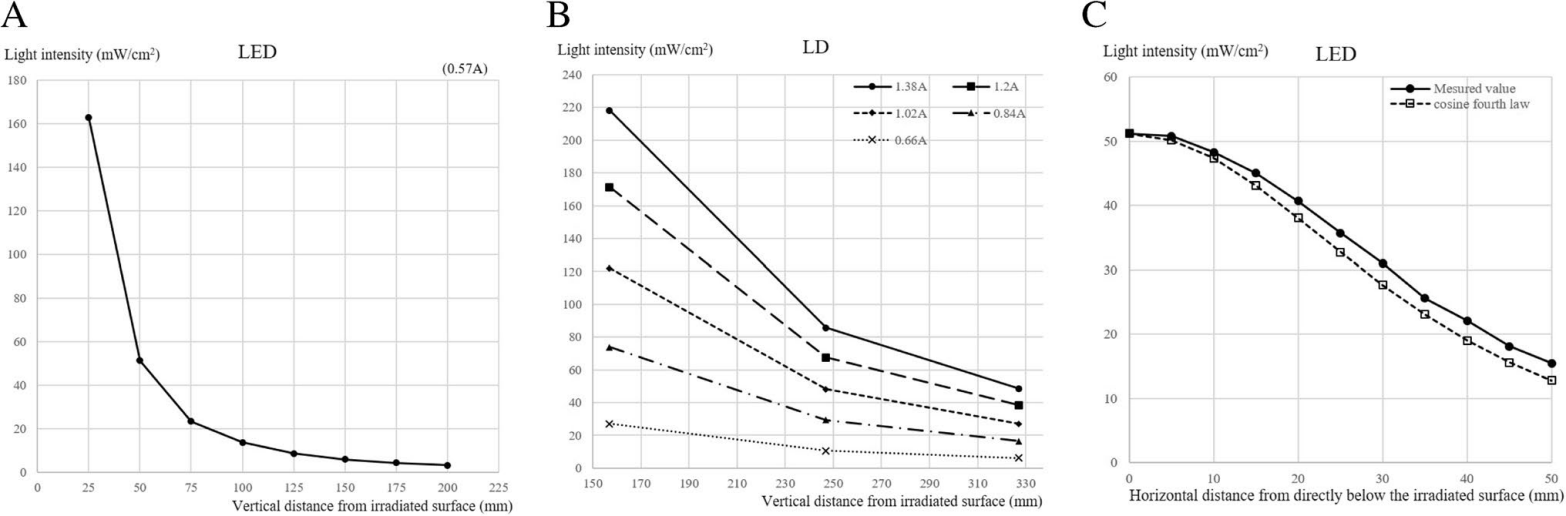
Light intensity as a function of vertical (A, B) and horizontal (C) distance from the area irradiated by the LED and LD systems

The light intensity measured directly below the irradiation surface was inversely proportional to the square of the vertical distance from the irradiated surface with both LED and LD devices (Fig. 3A, B).

The light irradiation intensity measured at different horizontal distances from directly below the surface irradiated by the LED device is shown in Fig. 3C. The dotted line in figure represents the cosine fourth law, which states that the illuminance on the plane at angle θ is cos4 θ times greater than the illuminance directly below the light source. The measured values agreed with the cosine quadrature law. Although the LD device has a narrow measurable range (40 × 40 mm) owing to its high linearity, the light irradiation intensity was equivalent at all locations within the measurable range.

### Effect of light irradiation on temperature

**Fig. 4:**
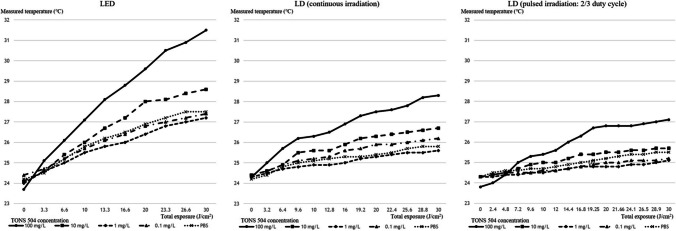
Temperature of samples following light irradiation

The temperature of the sample measured following light irradiation is shown in Fig. 4. With both devices in different irradiation modes, the temperature rise was high at high TONS concentrations. The temperature rise was the greatest during LED irradiation, followed by continuous LD irradiation and pulsed LD irradiation.

### Antimicrobial effect of TONS 504–aPDT

The antimicrobial effect of continuous irradiation (LED or LD, total irradiation of 30 J/cm^2^) in the presence of TONS 504 is shown in Table 1. Bacteria treated with either TONS 504 or light irradiation only manifested no growth inhibition. In contrast, bacteria exposed to 30 J/cm^2^ of LED or LD in the presence of > 0.1 mg/L TONS 504 underwent cell death, with no significant differences between the two groups.

**Table 1:** Number of colonies formed by bacteria in the three experimental groups

Experimental group	TONS 504 concentration (mg/L)				
	0	0.01	0.1	1	10
No irradiation	+++	+++	+++	+++	+++
LED (30 J/cm2)	+++	+++	135.7 ± 38.0	0	0
LD (30 J/cm2)	+++	+++	179.0 ± 53.1	0	0

Data are means ± SD from three independent experiments. +++ indicates > 300 CFU

**Fig. 5:**
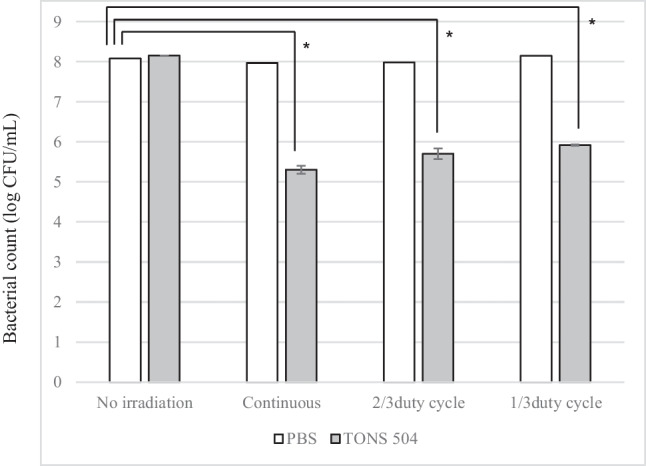
Anti-microbial effects of different LD irradiation modes. Quantification of *S. aureus* cell growth after TONS 504–aPDT. *P < 0.01 (one-way ANOVA followed by Dunnett’s post hoc test)

The anti-microbial effects of different LD irradiation modes are shown in Fig. 5. Bacteria treated with either TONS 504 (0.1 mg/L) or light irradiation only manifested no growth inhibition. There was no significant difference in the antimicrobial effect between aPDT by continuous LD irradiation and aPDT by pulsed LD irradiation. Notably, although the total light intensity of pulsed LD irradiation (20 J/cm^2^ at 2/3 duty cycle and 10 J/cm^2^ at 1/3 duty cycle) was smaller than that of continuous LD irradiation (total of 30 J/cm^2^), the antimicrobial effect was similar.

## Discussion

In this study, we compared two types of light irradiation devices for aPDT and found that the new LD light-irradiation device was more effective than the conventional LED light-irradiation device. aPDT requires high photosensitizer concentrations and intense light irradiation to achieve high antimicrobial efficacy. Mild conditions, such as low photosensitizer concentrations and low-power light irradiation, are desirable, and thus the choice of photosensitizer and irradiating light is important. We confirmed that, as a photosensitizer, TONS 504 exhibits a higher singlet oxygen quantum yield and photodynamic antibacterial effect than widely used methylene blue [15].

In this study, LD was used as a new light source, and the results showed that both LD and conventional LED light exhibited the usual light characteristics, namely the light intensity was inversely proportional to the square of the irradiation distance. LDs produce light that is directional because the coherent beam consists of waves in phase with each other. In contrast, LEDs emit light that is diffusely reflected because the photons are out-of-phase with each other [23]. Therefore, we characterized the diffused LED light, and the results showed that attenuation of the light intensity along the horizontal distance from the nadir of the LED light source obeyed the cosine quadrature law. Owing to the high directionality of LD light, there was no need to consider this form of light attenuation. Moreover, owing to the high optical linearity of LD light, the same light power as LED light was obtained even when the light source was sufficiently far away. Furthermore, the high directionality of LD light is an advantage because LD devices are designed to irradiate small organs, such as the cornea.

The new LD light-irradiation system used in this study was operated in the pulsed irradiation mode as well as the continuous irradiation mode. We expected pulsed irradiation to provide oxygen during the non-irradiation period. However, the anti-microbial effect of pulsed irradiation was not significantly different from that of continuous irradiation. Nevertheless, pulsed LD light irradiation resulted in the lowest temperature rise. The cornea is transparent and hemispherical, which allows light to pass through and form an image on the retina. Elevated temperatures have been reported to cause corneal collagen shrinkage [24, 25]. In particular, excessively high temperatures cause thermal denaturation of proteins, which decreases transparency and shape changes in the cornea, resulting in visual dysfunction.

The risk of transpupillary light exposure to the macula of the retina is high owing to the high directionality of LD light. Therefore, for in vivo applications, it is desirable to design a light irradiation system using LEDs, such as the system designed with an inclined ring-shaped irradiation surface to prevent light from entering the eye [21].

In conclusion, we demonstrated that the new LD light irradiation system provided the same anti-microbial effect but caused less damage than the conventional LED light irradiation system during TONS 504–aPDT. Further studies are thus warranted to evaluate the effect of TONS 504–aPDT on bacterial keratitis in vivo.
